# Intervening factors related to working conditions in nursing care quality

**DOI:** 10.1590/0034-7167-2025-0053

**Published:** 2025-12-08

**Authors:** João Vitor Silva Santos, Maithê de Carvalho e Lemos Goulart, Fernanda Maria Vieira Pereira-Ávila, Fernanda Garcia Bezerra Góes, Lucca Macedo dos Santos, Larissa Sousa Oliva Brun, Yanna Carla Pinheiro de Oliveira

**Affiliations:** I Universidade Federal Fluminense. Rio das Ostras, Rio de Janeiro, Brazil

**Keywords:** Workload, Working Conditions, Occupational Health, Nursing, Team, Quality of Health Care., Carga de Trabajo, Condiciones de Trabajo, Salud Laboral, Grupo de Enfermería, Calidad de la Atención de Salud.

## Abstract

**Objectives:**

to understand the intervening factors related to working conditions in nursing care quality.

**Methods:**

a qualitative study conducted between August and November 2024, with in-person interviews involving 35 nursing professionals from a public hospital in the coastal lowlands of Rio de Janeiro, Brazil. The data were processed using the *Interface de R pour les Analyses Multidimensionnelles de Textes et de Questionnaires* software and subjected to thematic analysis.

**Results:**

lack of supplies, work overload, insufficient organizational support, and poor infrastructure were the most frequently cited factors affecting working conditions and quality of care provided by nursing staff. The resulting classes highlight the structural, procedural, and outcome domains.

**Final Considerations:**

various factors related to working conditions were identified as intervening in nursing care quality, from the scarcity of supplies to professional development. Listening sessions can be effective in strengthening organizational support and implementing change.

## RESUMEN

**Objetivos:**

comprender los factores que influyen en las condiciones laborales en la calidad de la atención de enfermería.

**Métodos:**

estudio cualitativo realizado entre agosto y noviembre de 2024, mediante entrevistas personales con 35 profesionales de enfermería de un hospital público de la zona costera de Río de Janeiro, Brasil. Los datos se procesaron mediante el *software Interface de R pour les Analyses Multidimensionnelles de Textes et de Questionnaires* y se sometieron a análisis temático.

**Resultados:**

la falta de suministros, la sobrecarga de trabajo, el apoyo organizativo insuficiente y la infraestructura deficiente fueron los factores más frecuentemente citados que afectaron las condiciones laborales y la calidad de la atención brindada por el equipo de enfermería. Las clases resultantes destacan los dominios estructurales, procedimental y de resultados.

**Consideraciones Finales:**

se identificaron varios factores relacionados con las condiciones laborales que influyen en la calidad de la atención de enfermería, desde la escasez de suministros hasta el desarrollo profesional. Las sesiones de escucha pueden ser eficaces para fortalecer el apoyo organizacional e implementar el cambio.

## INTRODUCTION

Nursing professionals’ work focuses primarily on direct interaction with individuals under their care. Thus, the work performed by this team in healthcare services plays a central, ongoing role, directly related to quality of care, considering their significant representation in relation to other healthcare professionals^(1)^.

In Brazil, the inherent complexity of the work process for nursing teams working in secondary and tertiary healthcare creates challenges in providing quality care to meet patients’ needs. These challenges, combined with the often unfavorable context and working conditions faced by healthcare professionals, compromise not only quality of care provided but also workers’ health^(1)^.

Working conditions are defined as the set of factors involved in the execution of work activities. A work environment is characterized by several facets that can be adjusted and optimized, ranging from time management, working hours, rest periods, work schedules to dynamics related to professional categories and turnover^(2)^. This also includes remuneration aspects as well as the physical characteristics and mental demands present in the workplace. Professionals subjected to inadequate conditions, characterized by high demands, limited control over their functions, and scarce social support, face high risks of physical and psychological burnout, constituting a particularly vulnerable group. This scenario directly affects quality of care provided and patient safety^(2,3)^. As an example, a study carried out in hospital emergencies showed that the work overload faced by the nursing team, combined with understaffing, constitutes a preponderant factor in deterioration of quality of care, substantially increasing patient morbidity and mortality and significantly increasing adverse events related to healthcare^(4)^.

Healthcare quality can be understood as a care assessment that encompasses the analysis of structure, operational processes, and outcomes obtained. The structural dimension encompasses relatively stable elements, such as human resources, instruments, resource availability, facilities, and work organization models. The procedural dimension refers to activities performed by healthcare professionals in relation to patients, encompassing decision-making in the diagnostic, therapeutic, and preventive spheres. The outcome dimension is related to the effectiveness and efficiency of healthcare actions as well as the level of satisfaction expressed by clients^(5)^.

Therefore, it is a complex concept that varies according to the perspective of the person assessing it, be it a nurse, a patient/family or a nursing manager, and is assessed through indicators that are essential for managing best practices in hospital environments^(6-8)^. Some aspects are constantly identified as essential to quality of care, intervening in team satisfaction and professional retention, such as empathy, respect, patient advocacy and intentionality, which connect to nurses’ professionalism and commitment^(6,9)^.

In North American countries, such as Mexico, inadequate working conditions have a detrimental influence on quality of care, culminating in reduced resources allocated to staff and increased responsibilities assigned to workers in an undersized team^(10)^. It is important to note that excessive workloads, inadequate conditions, staff shortages, and a lack of resources can create an environment conducive to errors and service failures, jeopardizing patient safety and well-being. Therefore, investigating this relationship is crucial to identifying areas for improvement in nursing practice and resource management, both material and human, in public hospitals.

In this context, this study is justified by the need to understand nursing staff’s perceptions of their working conditions and the impact of these factors on quality of care provided. Giving voice to the workers directly involved in care is essential, as they possess the knowledge and experience necessary to identify areas requiring improvement, investment, or adjustments. From these professionals’ perspective, it becomes possible to contribute significantly to strengthening patient safety, improving quality of care, and achieving more efficient management. Furthermore, this approach can indirectly contribute to workers’ health by identifying priority paths to transforming working conditions that may currently be associated with compromising quality of care provided, especially in settings located in the countryside of the state under study. Furthermore, local studies can contribute as a source of contextualized evidence on practices, challenges, and solutions specific to a region or community.

Given the above, the study’s guiding question is: what factors related to working conditions interfere with nursing care quality?

## OBJECTIVES

To understand the intervening factors related to working conditions in nursing care quality.

## METHODS

### Ethical aspects

The research was approved by the Research Ethics Committee of the School of Humanities of the *Universidade Federal Fluminense* (Opinion 7,000,998 and Certificate of Presentation for Ethical Consideration 79329224.8.0000.8160), meeting the ethical requirements of Resolution 466/12 of the Brazilian National Health Council^(11)^. To ensure participant anonymity, the interviewer identified them by an alphanumeric code. All participants signed a written Informed Consent Form (ICF) and received an identical copy signed by the lead researcher.

### Study design

An exploratory study with a qualitative approach was conducted between August and November 2024 among nursing professionals working at a public hospital in a municipality located in the coastal lowlands of Rio de Janeiro state, Brazil. This study followed the COnsolidated criteria for REporting Qualitative Research recommendations^(12)^.

### Methodological procedures

In-person interviews were conducted using a semi-structured script developed specifically for this purpose and were led by a trained nursing undergraduate and the main researcher. Neither of these individuals had any close relationship with participants, thus ensuring that no judgment or bias was applied to their responses. Participants were informed that the interview would be recorded for later transcription and the exact moment the recording began. The interviews lasted a mean of 25 minutes.

The interviews followed a semi-structured script divided into two parts. The first part focused on participant characterization, containing closed-ended questions addressing sociodemographic and professional characteristics, such as age, race, religion, marital status, and education. To characterize the professional profile, questions about time since graduation, sector of activity, length of service at the hospital, whether a patient has more than one professional contract, and current weekly workload were used. The second part of the data collection instrument contains open-ended questions about the study’s objective, namely: what factors related to working conditions do you believe can affect nursing care quality in your work? Do you have specific examples of how these conditions have affected your ability to provide quality care? Could you share them?

### Study scenario

The hospital that forms the setting for this study is a medium-sized municipal institution and is the only public hospital in the municipality that serves an enrolled population of 156.491 inhabitants^(13)^. It has 271 nursing professionals, including 96 nurses, 147 nursing technicians and 28 nursing assistants, according to the National Registry of Health Establishments^(14)^. At this institution, nursing staff are governed by the municipal authority, and are mostly statutory and have limited-term employment contracts. The nursing staff work a 30-hour weekly workload, covering 24-hour shifts, plus additional work for the care team. It is worth noting that the institution regularly requests paid overtime from nursing staff.

### Sample

The sample consisted of nursing professionals who wished to participate in the study through convenience sampling, according to the following inclusion criteria: being a nurse, nursing technician, or nursing assistant who had worked at the hospital for at least six months. The exclusion criteria were nursing professionals who performed administrative functions or who were away from direct patient care, such as in examination departments or the materials center, as the aim was to understand the perspective of professionals who provide direct patient care. This study did not conduct a pilot test, as the interview question script is flexible and serves as a guide, not requiring standardized answers. Furthermore, the questions were understandable, and the responses achieved the objective.

### Data collection and organization

For data collection, professionals were approached during their work hours and invited to participate in the study. A convenient time, chosen by the professional, was awaited for the participant and the interviewer to go to a private room within their department to begin the consent process. This consisted of an explanation of the study’s objective and stages, reading the full text and signing the ICF. After signing the ICF, participants answered two questions: do you work in direct patient care? Do you perform only administrative functions in nursing? If a participant did not meet the eligibility criteria, their participation was terminated with a thank-you note for their availability. In this study, 49 professionals were approached, of whom 14 did not consent to participate, and there were no exclusions based on the eligibility criteria.

Once the study eligibility criteria were met, the interview began at a time determined by participants so as not to disrupt their work activities. During the data collection phase, it was anticipated that the recruitment of new participants would be interrupted if there was recurrent statements, repetition, or redundancy of content, which was considered theoretical data saturation^(15)^. However, for consistent data processing by *Interface de R pour les Analyses Multidimensionnelles de Textes et de Questionnaires* (IRAMUTEQ^®^), a minimum of 20 respondents is recommended^(15)^. Thus, from 20 participants, the study team began to observe data theoretical saturation, listening to the recordings and looking for the recurrence of the intervening factors mentioned by professionals without any new elements being added to their statements.

### Data analysis

Participant characterization data were compiled into an Excel^®^ spreadsheet for descriptive analysis, including absolute and relative frequencies and means. Interview recordings were transcribed and not returned to participants due to difficulty locating them in the study setting, as they were on-call staff. The transcriptions formed the text *corpus*, which was processed by IRAMUTEQ^®^ using word clouds, a similarity tree, and Descending Hierarchical Classification (DHC). Active forms of adjectives and nouns, adverbs, and verbs were considered, while other forms (conjunctions, pronouns, and prepositions) were considered supplementary forms, limited to a frequency of ten or more in the text *corpus*. For the similarity tree and DHC, adverbs were considered supplementary forms.

The higher the frequency of the word in the *corpus*, the more prominent it was in the word cloud^(16)^. The similarity tree portrayed the connectivity between the words in the text *corpus* ^(17)^, highlighting how the discursive content is structured through co-occurrence of words^(18)^. In DHC, lexical forms were grouped into classes using chi-square (χ2) test, which assesses the associative strength between word occurrence and classes. A χ2 value of 3.84 or greater and p<0.005 were considered, with emphasis on words with p<0.0001, which indicates a very strong association between the word and the class. DHC produced a dendrogram representing the various groupings and their most representative words^(18)^.

After processing qualitative data, thematic analysis was carried out, which aims to infer and interpret, discovering the structures of meaning that are part of communication, whose existence or repetition has meaning for the analysis^(19)^. The text *corpus* was organized and read in full to familiarize itself with the content and general ideas, in addition to understanding the text through a thorough reading of the text segments of the text *corpus*, observing the core meanings that elucidated nursing professionals’ perception based on the words highlighted in the word cloud, as well as the meaning of the connectivity between the branches in the similarity tree and, finally, the topics of each DHC class that emerged from the core meanings and were related to the conceptual dimensions of quality of care (structure, process and outcome).

## RESULTS

Thirty-five (100.0%) nursing professionals participated in the study, of which 13 (37.2%) were nurses and 22 (62.8%) were nursing technicians. The sample was composed mostly of women, with 33 (94.3%) female professionals and two (5.7%) males. The mean age was 40 years, ranging from 24 to 57 years. Concerning self-declared skin color, 14 (40.0%) identified themselves as white, 13 (37.1%) as brown, seven (20.0%) as black, and one (2.9%) as yellow. Of the total participants, 18 (51.4%) were married, 15 (42.9%) were single, and two (5.7%) were divorced.

Regarding training, 13 (37.1%) professionals had a technical degree, 11 (31.4%) had completed higher education, ten (28.6%) had a postgraduate degree, and one (2.9%) had a master’s degree. The time since training ranged from two to 27 years, with a mean of 14.5 years. The length of service at the hospital ranged from two to 22 years, with a mean of six years. Participants worked in different sectors: the internal medicine unit had ten (28.6%) participants, pediatrics, seven (20.0%), the surgical center, six (17.1%), and obstetrics, six (17.1%). Rooming-in had the participation of five (14.3%) professionals, and the Intensive Care Unit, one (2.9%).

A weekly workload of 40 hours was reported by 26 (74.3%) participants, who stated they had more than one employment relationship. Professionals reported spending a mean of 16 hours at the bedside during their shifts, ranging from four to 24 hours.

The text *corpus* resulting from professionals’ speeches was composed of 35 texts, presenting 26,141 occurrences of words, of which 2,654 were distinct words and 1,289 had a single occurrence (hapax), accounting for 4.9% of the word occurrences. The words most used by nursing professionals regarding working conditions and factors intervening in quality of care were “people” (f=495), “patient” (f=289), “being” (f=244), “time” (f=203), “relate” (f=155), “stay” (f=153), “thing” (f=142), “material” (f=127), “lack” (f=123), “example” (f=114), “assistance” (f=111), “give” (f=110), “end” (f=109), “work” (f=104) and “quality” (f=99).

**Figure 1:**
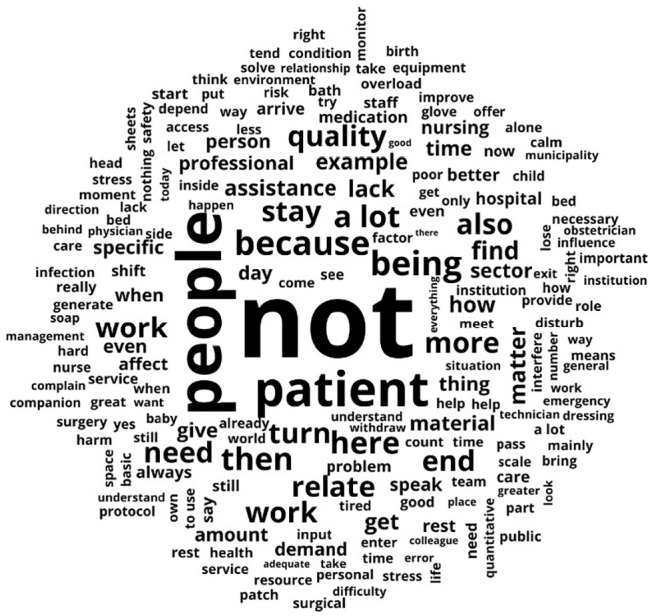
Text *corpus* word cloud on the perceptions of working conditions and factors intervening in quality of care of the nursing team working in a public hospital in the coastal lowlands of Rio de Janeiro, Rio das Ostras, Rio de Janeiro, Brazil, 2025

*Source: adapted from IRAMUTEQ^®^, 2025.*

The word cloud (Figure 1) shows the most frequently occurring words in the text *corpus*, with the word “no” standing out as nursing professionals’ main perception. This term reflects the absence of essential elements for quality care, highlighting structural and organizational gaps that directly impact professional practice. The emphasis on the word “no” highlights a lack of basic resources, such as supplies, adequate equipment, and appropriate physical conditions, essential for the proper performance of nursing activities.

Another aspect identified is the lack of organizational support, which contributes to the perception of devaluation of professionals, highlighting the difficulty in overcoming the challenges faced by nursing professionals without adequate management support.

The words “people” and “patient” stand out in the word cloud, reflecting the intrinsic relationship between nursing professionals and the object of their care. According to study participants, this relationship is profoundly affected by working conditions and insufficient nursing staff, resulting in an overload of tasks and reduced time dedicated to providing care to each patient. The rapid response times, a consequence of this overload, hinder the development of the bond necessary for implementing humanized practices.

**Figure 2:**
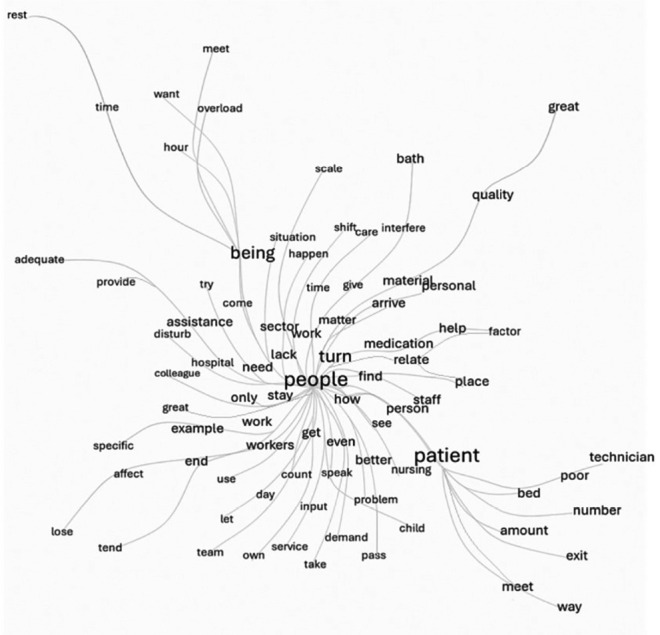
Similarity tree representing the connectivity between words about the perceptions of working conditions and factors affecting quality of care of the nursing team working in a public hospital in the coastal lowlands of Rio de Janeiro, Rio das Ostras, Rio de Janeiro, Brazil, 2025

*Source: adapted from IRAMUTEQ^®^, 2025.*

In the similarity tree (Figure 2), it was possible to verify that the words with the greatest connectivity are interconnected by thicker lines, appearing more prominently in the tree’s branches. Thus, in nursing professionals’ statements, a strong connectivity was revealed between the words “people”, in the center of the tree, and “being” and “patient”, in the main branches, reinforcing the word cloud assessment and revealing that nurses’ statements are permeated by words that establish the interpersonal relationship between them (the people) and their object of care (patients) in the context of nursing care.

The strong connection between the terms “people” and “being” highlights the perception that the constant presence of nursing professionals at patients’ bedside, with dedicated attention to prevent errors, is crucial. According to participants, this dynamic is seen as essential for providing quality care, as it involves continuous attention to patients’ needs, ensuring adequate care and preventing service failures, especially when it comes to medication administration and material shortages.

On the other hand, the word “patient”, in conjunction with “people”, and its co-occurrence with terms like “quantity” and “bed”, located in the more distal branches of the tree, reinforces the idea that nursing staff shortages are directly linked to the care provided. This finding highlights understaffing as a factor that impacts nursing care quality, compromising the ability to adequately meet patients’ needs.

**Figure 3:**
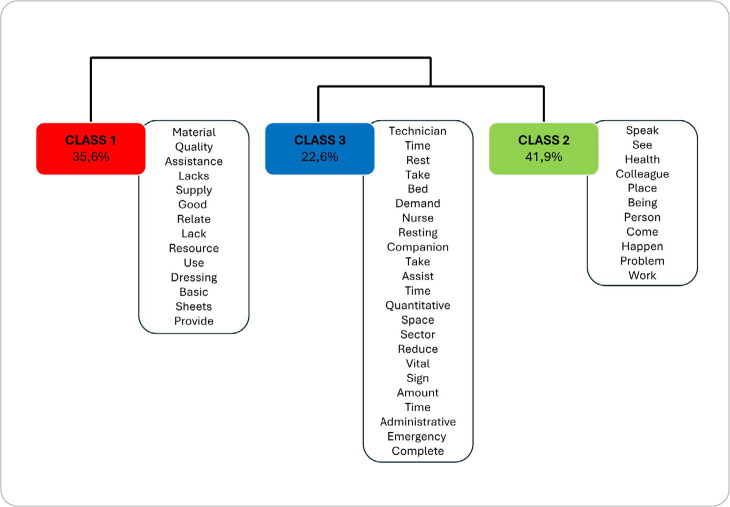
Descending Hierarchical Classification dendrogram, adapted from the *Interface de R pour les Analyses Multidimensionnelles de Textes et de Questionnaires* **, with significant words (p<0.0001) from the text** *corpus* on the perceptions of working conditions and factors intervening in quality of care of the nursing team working in a public hospital in the coastal lowlands of Rio de Janeiro, Rio das Ostras, Rio de Janeiro, Brazil, 2025

*Source: adapted from IRAMUTEQ^®^, 2025.*

In DHC analysis, 740 text segments were found, with 675 (91.2%) of them being classified into three distinct classes, representing excellent use of the text *corpus* (Figure 3). DHC dendrogram divided the *corpus* into two groups, of which one was subdivided into two other groups, totaling three classes, with class 2 comprising the largest percentage of text segments (41.9%).

The three classes generated by DHC were named and composed the following thematic units, which relate to quality of care dimensions: Structural dimension - Inputs and materials as intervening factors in nursing care quality; Procedural dimension - Nursing work conditions as intervening factors in nursing care quality; and Outcome dimension - Overload and precariousness of care as intervening factors in nursing care quality.

### Class 1: Structural dimension - Inputs and materials as intervening factors in nursing care quality

In class 1, the availability and quality of materials emerged as factors that influence quality of care, directly impacting nursing professionals’ daily practice in the study setting. The most frequently mentioned materials by participants were peripheral venous catheters, sutures, sheets, soap, gloves, and dressings. Professionals reported examples of inadequate or low-quality materials that malfunctioned, compromising both patient safety and work efficiency, highlighting the importance of having adequate resources for effective nursing performance.

*If there is no adequate material to make a dressing, to make a puncture, to make a suture in surgery, the assistance is compromised* [...]. (N04)

*Sometimes you end up losing your puncture because a polyfix doesn*’*t fit with a piece of equipment or a syringe breaks, so the quality of the material is essential for good work.* (NT05)

*Poor quality materials, sometimes gloves that don*’*t come in your size. You use a size small glove and only have a size large glove. There*’*s a risk there, and biosafety issues are also involved.* (N08)

Furthermore, it was observed that certain supplies are often completely lacking, forcing professionals to improvise solutions to provide care. This scenario poses risks for both the professional and the patient, in addition to resulting in greater physical and emotional exhaustion, intensifying stress in the workplace.

*There*’*s a lot missing; sometimes medication is available, sometimes it isn*’*t. We can*’*t rely on medication because some days it*’*s available at the institution and some days it isn*’*t.* (NT18)

[...] *so, you end up providing poor-quality service because you don*’*t have the materials you need. Another issue is the lack of supplies, especially when it comes to infection control.* (N05)

*It*’*s the lack of material* [...] *some basic things that would really help us provide better assistance and are not seen as a priority.* (NT03)

### Class 2: Procedural dimension - Nursing work conditions as intervening factors in nursing care quality

In class 2, nursing professionals’ statements reveal a worrying picture of precarious working conditions that impact both the health of these workers and quality of care provided to patients. Aspects such as a lack of organizational support, excessive workloads, and structural weaknesses in the healthcare system were recurring issues that generated dissatisfaction and negative repercussions.

*They wanted to fire me, but I refused and continued working like that, and the shifts took a toll on my health. During that time, I gained weight, became obese, developed a number of illnesses, didn*’*t see my family, and nearly had several accidents on the road.* (NT18)

*I*’*m sure people around me already say this. I recently had burnout, and the best thing that happened to me was changing industries, because it was really demanding, and I couldn*’*t do it. I completely changed.* (NT03)

*You can*’*t see a dirty room where no trash has been taken out and not speak up. If you*’*re there, in that environment, then you end up being responsible for resolving situations that aren*’*t your responsibility.* (N08)

Professionals’ statements highlighted the lack of organizational support as one of the main challenges they face on a daily basis. Furthermore, excessive workloads were identified as a constant factor in the statements, being cited as a significant interference in professionals’ quality of work and health.

*The workload could be shorter* [...] *it*’*s a bit complicated because healthcare is precarious these days throughout the state, and especially in the municipality.* (NT02)

[...] *the person goes to their boss, and your boss also sometimes doesn*’*t have the autonomy to talk to you or to help solve the problem.* (NT19)

Another point highlighted by the sample participants is the reassignment of professionals between departments within the institution. For professionals, this dynamic demonstrates a lack of recognition of their specific skills and the impact these changes have on their mental health and job performance.

*You come to work* [...] *I*’*m from the nursery, and I come to work in the nursery. You*’*re on the same shift, and out of nowhere you*’*re put in another department that has nothing to do with yours.* (NT02)

*We already come with that anxiety, and when it arrives, it*’*s really what*’*s going to happen* [...] *it*’*s stressful and they still demand too much from us.* (NT09)

*I*’*m not even talking about absence due to vacation, but rather completing the shift. It*’*s something management currently can*’*t resolve, and the infrastructure issue is no exception. We*’*re talking about nursing management.* (N10)

Nursing devaluation was perceived by nursing professionals, highlighting the lack of recognition and the low remuneration they receive as an intervening factor in quality of care provided.

*It*’*s not a bad place to work here. You need things in all the health units, but there*’*s a staff shortage, and the workload means you have to work elsewhere because they can*’*t even afford the minimum wage.* (N06)

*Nursing professionals aren*’*t valued very well. Often, you*’*re impatient with a patient because some people confuse their problems.* (NT19)

[...] *the workload in nursing. We have to work in several places. The problem isn*’*t just that; it*’*s the accumulation of work due to the low salary.* (NT12)

### Class 3: Outcome dimension - Overload and precariousness of care as intervening factors in nursing care quality

In class 3, nursing professionals’ reports highlight understaffing as a crucial factor in quality of care provided. The insufficient staff, highlighted by participants, overburdens the team, increasing the workload and compromising the ability to provide humane and safe care. Furthermore, the reassignment of professionals between departments is seen as a critical factor that destabilizes teams, undermines the continuity of care, and directly interferes with work organization.

*So, sometimes, the technician is taken from here to fill in for a break because of the other department*’*s break time, so they can take a break. We*’*re left with psychiatric patients and a shortage of professionals, and this hinders care delivery.* (NT08)

*I think the number of technicians working is lower than the number of patients we receive in our department. For example, here*’*s the emergency room* [...]. (N09)

*Having a nurse and a technician work with a high patient demand. I think the number of staff directly impacts quality of care, because you can*’*t be in all places at once.* (NT13)

*The number of staff is very small, and this ends up overloading their roles and compromising care. We end up not providing quality care. If they are overworked, it will impact quality of care and the patient*’*s prognosis. All of this will interfere with quality of care we provide.* (N04)

The lack of adequate rest periods emerges as a recurring problem, as reported by nursing professionals, who frequently highlight the difficulty of taking regular breaks during their work shifts. Their statements clearly show that a lack of rest negatively impacts professionals’ ability to concentrate and make decisions, resulting in more vulnerable and potentially unsafe care for patients.

*But to take your time off, you need a good space to take a shower, and you don*’*t have that. You won*’*t always have time to sleep.* (NT16)

*At midnight medication time, I can*’*t even tell what I*’*m doing anymore, because it*’*s been from eight in the morning until midnight without a break, without a lunch break, and sometimes without a break. There are only two technicians in the department.* (NT13)

*Because we had only one technician in the unit, a tent with 12 patients in the beds. Bedridden patients with enteral feeding tubes or urinary catheters... so we had to prioritize medication and vital signs.* (N07)

[...] *for this rest time, I think this would be essential, both for our psychology and for us to be able to rest and not have a time to rest. We get tired, and this tiredness sometimes gets in the way.* (NT08)

## DISCUSSION

The findings of this study revealed various factors that influence working conditions in nursing care quality, including insufficient material resources, low-quality materials, understaffing, lack of rest time, staff reassignment, work overload, and lack of organizational support. The lack of basic supplies and low-quality materials were directly perceived as factors that influence nursing care quality, as participating professionals resort to improvised solutions that can result in errors in professional practice and compromise the safety of care, according to statements.

A study conducted in two general hospitals in Iran, in Tehran, involving 211 nurses with at least two years of clinical experience, pointed to inadequate resources as a significant predictive factor for healthcare-associated infections and that it increases professionals’ stress levels, increasing the feeling of helplessness, data that supports the current findings^(20)^. Other studies are also in line with the findings of this study, highlighting the direct relationship between the lack of materials, the high workload and the precarious conditions faced by nursing professionals^(20,21)^.

In addition to insufficient supplies, excessive workload, work overload, and understaffing of nursing staff were shown to be factors that indirectly affect quality of care, as tired, overworked, and understaffed professionals cannot meet work demands, damaging their professional-patient relationship. A study in six Intensive Care Units with 105 nurses showed that long working hours, cumbersome workloads, and overload reduce the time for care provision, increasing the risk of errors, such as incorrect medication administration, patient falls, and hospital-acquired infections^(22)^, as mentioned by the participants in this study.

Similarly, research conducted among 810 hospital nurses in the United States of America, working at least 36 hours per week, revealed that work overload compromises professionals’ health and patient safety, highlighting the need for organizational interventions to improve staff allocation and distribution of functions^(23)^. Nursing understaffing compromises technical efficiency, professionals’ ethical responsibilities, and quality of care. A study in the Philippines found that workload and lack of resources exacerbate the problem, requiring structural reforms in the healthcare system. The high patient-to-nurse ratio intensifies the overload, contributing to dissatisfaction, burnout, and adverse events, in addition to reducing staff satisfaction^(21)^. This scenario is similar to that perceived by participants in this research.

An insufficient number of professionals results in an overload of work that often leads to the omission of essential care. According to COFEN Resolution 543/2017, adequate staffing is essential to ensure safe and quality care, and is nurses’ exclusive responsibility^(24)^. The results of research carried out in three Intensive Care Units of hospitals in Brazil with 29 nursing professionals are in line with the findings of this study, indicating that understaffing generates several repercussions, including negative impacts on quality of care provided and on worker satisfaction^(25)^. Therefore, adequately sizing the nursing team is essential to balance workload and improve working conditions.

The findings of this research highlight that lack of rest, combined with long workloads, work overload, and understaffing, impacts quality of care. Rest for professionals is a constitutional right, guaranteed at least one hour for workdays longer than six hours, two hours for 12-hour workdays, and four hours for 24-hour workdays, with the possibility of splitting the rest period, as per collective bargaining agreements^(26)^. It is essential, therefore, that the rest conditions provided for by law are met, with adequate, air-conditioned, acoustically isolated places proportional to the number of professionals, both in public and private institutions, ensuring comfort, dignity and reducing the impacts of long working hours^(27)^.

Lack of rest among nursing professionals contributes to adverse health events. A study of 588 nurses in China showed that poor sleep quality and lack of adequate rest compromise judgment, memory, and attention, increasing the risk of errors in environments requiring high concentration and rapid decision-making, especially in environments requiring high concentration and rapid decision-making, such as healthcare settings^(28)^.

Besides rest, another frequently mentioned factor was nursing staff reassignment, a recurring issue at the institution due to understaffing. Nursing professionals feel undervalued, which generates dissatisfaction and affects quality of care. Structural problems, low wages, and lack of recognition contribute to high turnover and weakened resilience. Thus, the perception of undervaluation, coupled with a lack of autonomy, reinforces the need for greater management support to strengthen the team^(29)^.

Lack of organizational support and lack of dialogue with leadership were widely cited as aggravating factors in working conditions. This fact, as is the case in this study, was also reported in a study conducted in general hospitals in Iran, showing that insufficient resources, combined with poor leadership and the exclusion of nurses from organizational decisions, compromise both staff satisfaction and quality of care, creating a stressful environment^(20)^.

Thus, it is highlighted that organizational support is crucial to deal with the shortage of human resources, materials and inputs, in addition to improving workflows and interpersonal relationships^(29)^. The lack of this support leads to dissatisfaction, work overload, and increased absenteeism, negatively impacting the care provided and team motivation. Organizational support is cited as a key aspect of motivation, performance, and the quality of services provided, in addition to directly impacting employee satisfaction.

### Study limitations

Limitations are associated with the sensitive nature of the topic, which led to several refusals from nursing professionals. This resistance may have been influenced by concerns about confidentiality and the perception of potential repercussions on working conditions and relationships with management. Despite this, it was possible to achieve data theoretical saturation, with a representative sample of nursing professionals working in the investigated setting. Furthermore, data collection was restricted to a single public hospital, which may limit the generalizability of the findings to other contexts and healthcare institutions.

### Contributions to nursing, health or public policy

In hospital environments, institutional support and nursing leadership are crucial to address resource scarcity, improve interpersonal relationships, and optimize workflows. This study highlights the urgency of structural changes in the healthcare system to protect professionals’ and patients’ health. Measures such as regulatory board oversight, payment of minimum wages, adequate staffing, guaranteed rest, and strengthening leadership are essential to reverse this situation. Therefore, it is understood that nursing professionals’ well-being is essential to ensure not only the safety and quality of care, but also healthcare system sustainability. These efforts, when incorporated effectively, can significantly transform quality of care, clinical outcomes, and professionals’ experience in hospital environments.

## FINAL CONSIDERATIONS

Working conditions, such as the lack of adequate rest periods and places, frequent reassignments to other departments, low wages, professional devaluation, and lack of institutional support, directly impact nursing care quality. Furthermore, nursing professionals perceive shortages of materials and supplies, work overload, and understaffing as contributing factors.

The study offers important contributions to nursing practice by fostering critical reflection among nursing leaders and managers, fostering strategies that foster improved working conditions and, consequently, quality of care provided. Therefore, the results point to the need to implement organizational changes that promote professional appreciation, ensure team well-being, and ensure adequate working conditions. Hence, viable strategies to strengthen organizational support, improve internal communication, and foster a collaborative environment include holding regular meetings, listening sessions, and collective dialogue sessions. These actions can serve as tools for ongoing mapping of the institutional landscape, providing concrete support for formulating and implementing changes consistent with the reality of services.

Furthermore, the study highlights the State’s responsibility as employer, manager, and regulator of public health services, ensuring adequate working conditions for teams. Failure to fulfill this role contributes to the perpetuation of structural and human problems that compromise not only quality of care but also the nursing staff’s physical and mental health, directly impacting patient safety. Thus, the study’s findings provide evidence that can inform public policies aimed at restructuring nursing work, professional development, and strengthening care, promoting sustainable transformations that ensure the quality of services offered and workers’ health.

Finally, we suggest that future implementation research be conducted, especially in nursing management, focusing on assessing indicators related to working conditions before and after the adoption of priority institutional interventions. Studies that consider workers’ active participation and include an analysis of the impact of the changes adopted could strengthen the applicability of the findings and contribute to the continuous improvement of nursing care quality and the organization of work in health services.

## Data Availability

The research data are available only upon request.
