# Supplementary material for: Intervening factors related to working conditions in nursing care quality
Source: Rev Bras Enferm. 2025 Dec 8;78(6):e20250053. doi: 10.1590/0034-7167-2025-0053 (PMC12707608; doi:10.1590/0034-7167-2025-0053)
Supplement: Translation

# Translation: Fatores intervenientes relacionados às condições de trabalho na qualidade da assistência de enfermagem

**DOI:** 10.1590/0034-7167-2025-0053pt

**Authors:** João Vitor Silva Santos, Maithê de Carvalho e Lemos Goulart, Fernanda Maria Vieira Pereira-Ávila, Fernanda Garcia Bezerra Góes, Lucca Macedo dos Santos, Larissa Sousa Oliva Brun, Yanna Carla Pinheiro de Oliveira

**Affiliations:** I Universidade Federal Fluminense. Rio das Ostras, Rio de Janeiro, Brasil

**Keywords:** Carga de Trabalho, Condições de Trabalho, Saúde do Trabalhador, Equipe de Enfermagem, Qualidade da Assistência à, Saúde.

## Abstract

**Objetivos:**

compreender os fatores intervenientes relacionados às condições de trabalho na qualidade da assistência de enfermagem.

**Métodos:**

estudo qualitativo, realizado entre agosto e novembro de 2024, com entrevistas presenciais envolvendo 35 profissionais de enfermagem de um hospital público da baixada litorânea do Rio de Janeiro, Brasil. Os dados foram processados pelo *software Interface de R pour les Analyses Multidimensionnelles de Textes et de Questionnaires* e submetidos à Análise Temática.

**Resultados:**

falta de insumos, sobrecarga de trabalho, apoio organizacional insuficiente e infraestrutura precária foram fatores intervenientes relacionados às condições de trabalho na qualidade da assistência mais citados pela equipe de enfermagem. As classes geradas destacam os domínios estrutural, processual e de resultado.

**Considerações Finais:**

diversos fatores das condições de trabalho foram apontados como intervenientes na qualidade da assistência de enfermagem, precariedade de insumos até a valorização profissional. Momentos de escuta podem ser eficazes para fortalecer apoio organizacional e implementar mudanças.

## INTRODUÇÃO

A atuação laboral dos profissionais de enfermagem se concentra principalmente na interação direta com os indivíduos sob seus cuidados. Assim, o trabalho realizado por esta equipe nos serviços de saúde desempenha um papel central, contínuo e diretamente relacionado à qualidade da assistência, considerando sua expressiva representatividade em relação aos demais profissionais da área da saúde^(1)^.

No Brasil, a complexidade inerente ao processo laboral da equipe de enfermagem que atua na prestação de cuidados de saúde de nível secundário e terciário traz desafios na oferta de assistência de qualidade para atender às necessidades dos pacientes. Esses desafios, somados ao contexto e condições de trabalho muitas vezes desfavoráveis, enfrentados pelos profissionais de saúde, comprometem não apenas a qualidade da assistência prestada, mas também a saúde do trabalhador^(1)^.

As condições de trabalho são descritas como o conjunto de fatores que envolvem a execução das atividades laborais. Um ambiente laboral é caracterizado por diversas facetas que podem ser ajustadas e otimizadas, abrangendo desde a gestão do tempo, horas de trabalho, períodos de descanso, escalas de trabalho até as dinâmicas relacionadas às categorias profissionais e à rotatividade^(2)^. Isso inclui, ainda, aspectos remunerativos, bem como as características físicas e demandas mentais presentes no local de trabalho. Profissionais submetidos a condições inadequadas, caracterizadas por altas demandas, controle limitado sobre suas funções e escasso apoio social, enfrentam riscos elevados de desgaste físico e psicológico, configurando-se em um grupo particularmente vulnerável. Esse cenário afeta diretamente a qualidade da assistência prestada e a segurança do paciente^(2,3)^. Como exemplo, estudo realizado em emergências hospitalares evidenciou que a sobrecarga de trabalho enfrentada pela equipe de enfermagem, combinada com o subdimensionamento de pessoal, constitui um fator preponderante na deterioração da qualidade da assistência, aumentando substancialmente a morbimortalidade dos pacientes e significativamente eventos adversos relacionados à assistência em saúde^(4)^.

A qualidade da assistência em saúde pode ser entendida como uma avaliação assistencial que compreende a análise de estrutura, processos operacionais e resultados obtidos. A dimensão estrutural abarca os elementos relativamente estáveis, como recursos humanos, instrumentos, disponibilidade de recursos, instalações e modelos de organização do trabalho. A dimensão processual refere-se às atividades executadas pelos profissionais de saúde em relação aos pacientes, englobando a tomada de decisão nos âmbitos diagnóstico, terapêutico e preventivo. A dimensão de resultado está relacionada à efetividade e eficiência das ações em saúde, bem como ao nível de satisfação manifestado pelos clientes^(5)^.

Trata-se, portanto, de conceito complexo que varia conforme a perspectiva de quem a avalia, seja o enfermeiro, o paciente/família ou o gestor de enfermagem, sendo avaliado por meio de indicadores indispensável para gestão das melhores práticas no ambiente hospitalar^(6-8)^. Alguns aspectos são constantemente identificados como essenciais à qualidade da assistência, intervindo na satisfação da equipe e retenção dos profissionais, como empatia, respeito, defesa do paciente e intencionalidade, que se conectam ao profissionalismo e comprometimento dos enfermeiros^(6,9)^.

Em países da América do Norte, como o México, as condições de trabalho inadequadas exercem influência prejudicial na qualidade da assistência, culminando em redução dos recursos destinados à equipe e acréscimo nas responsabilidades atribuídas aos trabalhadores em uma equipe subdimensionada^(10)^. Cabe destacar que cargas horárias excessivas, condições inadequadas, escassez de pessoal e falta de recursos podem criar um ambiente propício a erros e falhas no atendimento, colocando em risco a segurança e o bem-estar dos pacientes. Portanto, investigar essa relação é crucial para identificar áreas de melhoria na prática de enfermagem e na gestão de recursos, materiais e humanos em hospitais públicos.

Nesse contexto, este estudo justifica-se pela necessidade de compreender as percepções da equipe de enfermagem sobre as condições de trabalho nas quais estão inseridos e os impactos desses fatores na qualidade da assistência prestada. Dar voz aos trabalhadores que atuam diretamente na assistência é essencial, pois são eles que possuem o conhecimento e a vivência necessários para identificar áreas que demandam aprimoramento, investimento ou ajustes. A partir da ótica desses profissionais, torna-se possível colaborar de maneira significativa para fortalecimento da segurança do paciente, melhoria da qualidade da assistência e uma gestão mais eficiente. Além disso, essa abordagem pode contribuir, de forma indireta, para a saúde dos próprios trabalhadores, ao apontar caminhos prioritários para transformar condições de trabalho que, atualmente, podem estar associadas ao comprometimento da qualidade do cuidado oferecido, especialmente em cenários localizados no interior do estado em estudo. Ademais, estudos locais podem contribuir como fonte de evidência contextualizada sobre práticas, desafios e soluções específicas de uma região ou comunidade.

Diante do exposto tem-se como questão norteadora do estudo: quais fatores relacionados às condições de trabalho interferem na qualidade da assistência de enfermagem?

## OBJETIVOS

Compreender os fatores intervenientes relacionados às condições de trabalho na qualidade da assistência de enfermagem.

## MÉTODOS

### Aspectos éticos

A pesquisa foi aprovada pelo Comitê de Ética em Pesquisa da Faculdade de Humanas da Universidade Federal Fluminense (Parecer n^o^ 7.000.998 e Certificado de Apresentação para Apreciação Ética n^o^ 79329224.8.0000.8160), atendendo às exigências éticas da Resolução n^o^ 466/12 do Conselho Nacional de Saúde^(11)^. Para garantir o anonimato dos participantes, o entrevistador chamou-os por um código de identificação alfanumérico. Todos os participantes assinaram, por escrito, o Termo de Consentimento Livre e Esclarecido (TCLE) e receberam uma via, de igual teor, assinada pela pesquisadora principal.

### Tipo de estudo

Estudo exploratório, com abordagem qualitativa, realizado entre agosto e novembro de 2024 entre profissionais de enfermagem que atuam em um hospital público de um município situado na baixada litorânea do estado do Rio de Janeiro, Brasil. Este estudo seguiu as recomendações do *COnsolidated criteria for REporting Qualitative research* ^(12)^.

### Procedimentos metodológicos

As entrevistas presenciais foram realizadas utilizando um roteiro semiestruturado, desenvolvido para este fim, e foram conduzidas por um graduando de enfermagem, devidamente capacitado e pela pesquisadora principal. Ambos não possuem qualquer relação de proximidade com os participantes, garantindo, assim, a isenção de julgamentos ou vieses nas respostas. Foram informados ao participante que a entrevista seria gravada para posterior transcrição e o exato momento em que a gravação iniciou. As entrevistas tiveram duração média de 25 minutos.

As entrevistas seguiram um roteiro semiestruturado dividido em duas partes, sendo a primeira para caracterização do participante, contendo questões fechadas abordando a caracterização sociodemográfica e profissional, como idade, cor da pele, religião, estado civil e escolaridade. Para caracterização do perfil profissional, foram utilizadas perguntas sobre tempo de formação, setor de atuação, tempo de atuação no referido hospital, se possui mais de um vínculo profissional e carga horária semanal atual. A segunda parte do instrumento de coleta de dados possui questões abertas sobre o objetivo do estudo, a saber: quais os fatores relacionados às condições de trabalho que você acha que podem intervir na qualidade da assistência de enfermagem no seu trabalho? Você tem exemplos específicos de como as condições afetaram a sua capacidade de proporcionar uma assistência de qualidade? Poderia compartilhar?

### Cenário do estudo

O hospital que compõe o cenário deste estudo é uma instituição de autarquia municipal com médio porte, e é o único hospital público do município que atende a uma população adscrita de 156.491 habitantes^(13)^. Conta com 271 profissionais de enfermagem, sendo 96 enfermeiros, 147 técnicos de enfermagem e 28 auxiliares de enfermagem, de acordo com o Cadastro Nacional dos Estabelecimentos de Saúde^(14)^. Na referida instituição, os vínculos empregatícios dos trabalhadores de enfermagem são regidos pela autarquia municipal, sendo estatutários majoritariamente e com contratos de trabalho com tempo limitado. A carga horária semanal de trabalho da enfermagem é de 30 horas, realizadas em plantão de 24 horas semanais, além de trabalho complementar para a equipe assistencial. Ressalta-se que, de forma recorrente, a instituição solicita horas extras remuneradas aos trabalhadores de enfermagem.

### Amostra

A amostra foi composta por profissionais de enfermagem que desejaram participar do estudo em uma amostragem por conveniência, de acordo com os seguintes critérios de inclusão: ser enfermeiros, técnicos ou auxiliares de enfermagem atuantes há pelo menos seis meses no hospital. Os critérios de exclusão foram ser profissionais de enfermagem que exerciam funções administrativas ou que estavam afastados da assistência direta aos pacientes, como em setores de exames ou central de materiais, uma vez que buscou-se compreender a ótica de profissionais que prestam assistência direta ao paciente. Neste estudo, não foi realizado teste piloto, pois o roteiro de perguntas em uma entrevista é flexível e serve como um guia, não requerendo respostas padronizadas. Ademais, as perguntas foram compreensíveis e as respostas alcançaram o objetivo.

### Coleta e organização dos dados

Para a coleta de dados, os profissionais foram abordados em sua jornada de trabalho e convidados a participar da pesquisa. Foi aguardado um momento oportuno, escolhido pelo profissional, para que o participante e o entrevistador se dirigissem a uma sala reservada no próprio setor de atuação para iniciar o processo de consentimento, que consistiu na explicação do objetivo e etapas do estudo, com leitura na íntegra e assinatura do TCLE. Após assinatura do TCLE, o participante respondeu a duas perguntas, sendo: você atua na assistência direta ao paciente? Você exerce somente funções administrativas na enfermagem? Caso o participante não atendesse aos critérios de elegibilidade, sua participação seria encerrada com um agradecimento pela disponibilidade. Neste estudo, foram abordados 49 profissionais, dos quais 14 não consentiram sua participação, e não houve exclusões pelos critérios de elegibilidade.

Uma vez atendidos os critérios de elegibilidade para o estudo, a entrevista foi iniciada no momento oportuno definido pelo participante de forma que não atrapalhasse suas atividades laborais. Foi previsto que, durante a etapa de coleta de dados, houvesse interrupção da captação de novos participantes caso houvesse a recorrência das falas, repetição ou redundância dos conteúdos, sendo compreendido como saturação teórica dos dados^(15)^. Entretanto, para o consistente processamento de dados pelo *software Interface de R pour les Analyses Multidimensionnelles de Textes et de Questionnaires* (IRAMUTEQ^®^), é indicada a captação mínima de 20 entrevistados^(15)^. Assim, a partir de 20 participantes, a equipe do estudo passou a observar a saturação teórica dos dados, fazendo a escuta das gravações e buscando a recorrência dos fatores intervenientes citados pelos profissionais sem que houvesse nenhum novo elemento acrescentado em suas falas.

### Análise dos dados

Os dados de caracterização dos participantes constituíram uma planilha do *software Excel* ^®^ para análise descritiva, como frequência absoluta e relativa e média. As gravações das entrevistas foram transcritas e não foram devolvidas aos participantes, pela dificuldade de encontrá-los no cenário do estudo, uma vez que são plantonistas. A transcrição formou o *corpus* textual, que foi processado pelo *software* IRAMUTEQ^®^, por meio da nuvem de palavras, pela árvore de similitude e pela Classificação Hierárquica Descendente (CHD). Foram consideradas as formas ativas de adjetivos e substantivos, advérbios e verbos, e as demais formas (conjunções, pronomes e preposições) como formas suplementares, limitados a frequência de dez vezes ou mais no *corpus* textual. Para a árvore de similitude e CHD, os advérbios foram considerados como forma suplementar.

Foi considerada a maior frequência do vocábulo no *corpus*, quanto maior destaque o vocábulo apresentasse na Nuvem de Palavras^(16)^. A árvore de similitude retratou a conexidade entre as palavras do *corpus* textual^(17)^, evidenciando como se estrutura o conteúdo discursivo por meio da coocorrência dos vocábulos^(18)^. Na CHD, houve o agrupamento das formas lexicais em classes, realizado a partir do teste qui-quadrado (χ2), que verifica a força associativa entre a ocorrência das palavras e as classes. Consideraram-se o χ2 igual ou superior a 3,84 e p<0,005, com destaque aos vocábulos com p<0,0001, que denota associação fortíssima do vocábulo na classe. A CHD produziu um dendrograma que representa os diversos grupamentos e suas palavras mais representativas^(18)^.

Após o processamento dos dados qualitativos, foi realizada a análise temática, que se propõe à inferência e interpretação, descobrindo as estruturas de sentido que fazem parte da comunicação, cuja existência ou repetição tenha significado para a análise^(19)^. Realizaram-se organização e leitura na íntegra do *corpus* textual, para a familiarização com o conteúdo e ideias gerais, além da compreensão do texto por meio da leitura minuciosa dos segmentos de texto do *corpus* textual, observando os núcleos de sentido que elucidaram a percepção dos profissionais de enfermagem a partir das palavras em destaque na nuvem de palavras, assim como o significado da conexidade entre os ramos na árvore de similitude e, por fim, os temas de cada classe da CHD que emergiram dos núcleos de sentido e se relacionaram às dimensões conceituais da qualidade da assistência (estrutura, processo e resultado).

## RESULTADOS

Participaram do estudo 35 (100,0%) profissionais de enfermagem, sendo 13 (37,2%) enfermeiros e 22 (62,8%) técnicos de enfermagem. A amostra foi composta majoritariamente por mulheres, sendo 33 (94,3%) profissionais do sexo feminino e dois (5,7%) do sexo masculino. A média de idade foi de 40 anos, variando entre 24 e 57 anos. Em relação à autodeclaração de cor da pele, 14 (40,0%) identificaram-se como brancos, 13 (37,1%), pardos, sete (20,0%), pretos e um (2,9%), amarelo. Do total de participantes, 18 (51,4%) eram casados, 15 (42,9%), solteiros e dois (5,7%), divorciados.

No âmbito da formação, 13 (37,1%) profissionais possuíam nível técnico, 11 (31,4%), ensino superior completo, dez (28,6%), pós-graduação e um (2,9%), mestrado. O tempo de formação variou entre dois e 27 anos, com média de 14,5 anos. O tempo de atuação no hospital variou entre dois e 22 anos, com média de seis anos. Os participantes atuavam em diferentes setores, sendo a unidade de clínica médica com dez (28,6%) participantes, pediatria, com sete (20,0%), centro cirúrgico, com seis (17,1%) e obstetrícia, com seis (17,1%). O alojamento conjunto contou com a participação de cinco (14,3%) profissionais e a Unidade de Terapia Intensiva, com um (2,9%).

A carga horária semanal de 40 horas foi relatada por 26 (74,3%) participantes, que afirmaram possuir mais de um vínculo empregatício. Os profissionais afirmaram dedicar 16 horas em média na beira do leito durante os plantões, variando entre quatro e 24 horas.

O *corpus* textual advindo das falas dos profissionais foi composto por 35 textos, apresentando 26.141 ocorrências de palavras, sendo 2.654 palavras distintas e 1.289 com uma ocorrência única (hápax), representando 4,9% das ocorrências de palavras. As palavras mais utilizadas pelos profissionais de enfermagem sobre as condições de trabalho e fatores intervenientes na qualidade da assistência foram “gente” (f=495), “paciente” (f=289), “estar” (f=244), “vez” (f=203), “relacionar” (f=155), “ficar” (f=153), “coisa” (f=142), “material” (f=127), “falta” (f=123), “exemplo” (f=114), “assistência” (f=111), “dar” (f=110), “acabar” (f=109), “trabalhar” (f=104) e “qualidade” (f=99).

Na nuvem de palavras (Figura 1), é possível visualizar as palavras mais recorrentes no *corpus* textual, e em posição de centralidade está o vocábulo “não”, destacando-se como a principal percepção dos profissionais de enfermagem. Esse termo reflete a ausência de elementos essenciais para a assistência de qualidade, apontando lacunas estruturais e organizacionais que impactam diretamente a prática profissional. O destaque da palavra “não” evidencia um cenário de carência de recursos básicos, como insumos, equipamentos adequados e condições físicas apropriadas, fundamentais para o desempenho adequado das atividades de enfermagem.

**Figura 1:** Nuvem de palavras do *corpus* textual sobre as percepções das condições de trabalho e fatores intervenientes na qualidade da assistência da equipe de enfermagem atuante em um hospital público da baixada litorânea do Rio de Janeiro, Rio das Ostras, Rio de Janeiro, Brasil, 2025

*Fonte: adaptado do IRAMUTEQ^®^, 2025.*

Outro aspecto identificado é a insuficiência de apoio organizacional, que contribui para a percepção de desvalorização dos profissionais, evidenciando a dificuldade em superar os desafios enfrentados pelos profissionais de enfermagem sem o suporte adequado da gestão.

Os vocábulos “gente” e “paciente” destacam-se na nuvem de palavras, refletindo a relação intrínseca entre o profissional de enfermagem e o objeto de seu cuidado. De acordo com os participantes do estudo, esse binômio é profundamente afetado pelas condições de trabalho e insuficiência de profissionais de enfermagem, o que resulta em uma sobrecarga de tarefas, reduzindo o tempo dedicado na assistência para cada paciente. Os atendimentos rápidos, consequência dessa sobrecarga, dificultam o desenvolvimento do vínculo necessário para a implementação de práticas humanizadas.

Foi possível verificar que, na árvore de similitude (Figura 2), os vocábulos que possuem maior conexidade entre si são interligados por linhas mais espessas, aparecendo em maior destaque nos ramos da árvore. Revelou-se, então, nas falas dos profissionais de enfermagem a forte conexidade entre os vocábulos “gente”, no centro da árvore, e “estar” e “paciente”, nos ramos principais, reforçando a avaliação da nuvem de palavras e revelando que as falas dos profissionais de enfermagem estão permeadas pelos vocábulos que estabelecem a relação interpessoal entre eles (a gente) e o seu objeto de cuidado (pacientes) no contexto da assistência de enfermagem.

**Figura 2:** Árvore de similitude representando a conexidade entre as palavras sobre as percepções das condições de trabalho e fatores intervenientes na qualidade da assistência da equipe de enfermagem atuante em um hospital público da baixada litorânea do Rio de Janeiro, Rio das Ostras, Rio de Janeiro, Brasil, 2025

*Fonte: adaptado do IRAMUTEQ^®^, 2025.*

A forte conexidade entre os termos “gente” e “estar” destaca a percepção de que é fundamental a presença constante dos profissionais de enfermagem ao lado do paciente, com atenção dedicada para evitar erros. De acordo com as falas dos participantes, essa dinâmica é vista como essencial para a prestação de uma assistência de qualidade, pois envolve a atenção contínua às necessidades dos pacientes, garantindo cuidados adequados e prevenindo falhas no atendimento, sobretudo quando se referem à administração de medicamentos e à falta de materiais.

Por outro lado, o vocábulo “paciente”, em conexidade com “gente”, além de sua coocorrência com termos como “quantidade” e “leito”, localizados nos ramos mais distais da árvore, reforça a ideia de que a escassez de pessoal de enfermagem está diretamente associada ao cuidado prestado. Esse achado evidencia o subdimensionamento como um fator interveniente na qualidade da assistência de enfermagem, comprometendo a capacidade de atendimento adequado às necessidades dos pacientes.

Na análise da CHD, foram encontrados 740 segmentos de texto, com 675 (91,2%) deles sendo classificados em três classes distintas, representando excelente aproveitamento do *corpus* textual (Figura 3). O dendrograma da CHD dividiu o *corpus* em dois grupos, no qual um deles se subdividiu em outros dois grupos, totalizando três classes, sendo a classe 2 composta pelo maior percentual dos segmentos de texto (41,9%).

**Figura 3:** Dendrograma da Classificação Hierárquica Descendente, adaptado do *Interface de R pour les Analyses Multidimensionnelles de Textes et de Questionnaires* **, com palavras significativas (p<0,0001) do** *corpus* textual sobre as percepções das condições de trabalho e fatores intervenientes na qualidade da assistência da equipe de enfermagem atuante em um hospital público da baixada litorânea do Rio de Janeiro, Rio das Ostras, Rio de Janeiro, Brasil, 2025

*Fonte: adaptado do IRAMUTEQ^®^, 2025.*

As três classes geradas pela CHD foram nomeadas e compuseram as seguintes unidades temáticas, que se relacionam com as dimensões da qualidade da assistência: Dimensão estrutural - Insumos e materiais como fatores intervenientes na qualidade da assistência de enfermagem; Dimensão processual - As condições do trabalho da enfermagem como fatores intervenientes à qualidade da assistência de enfermagem; e Dimensão resultado - A sobrecarga e precarização do cuidado como fatores intervenientes na qualidade da assistência de enfermagem.

### Classe 1: Dimensão estrutural - Insumos e materiais como fatores intervenientes na qualidade da assistência de enfermagem

Na classe 1, a disponibilidade e a qualidade dos materiais emergiram como fatores intervenientes na qualidade da assistência, impactando diretamente a prática diária dos profissionais de enfermagem no cenário do estudo. Os insumos materiais mais mencionados pelos participantes foram cateter venoso periférico, fio de sutura, lençol, sabão, luva e curativo. Os profissionais relataram exemplos de materiais inadequados ou de baixa qualidade que não funcionaram corretamente, comprometendo tanto a segurança do paciente quanto a eficiência do trabalho, destacando a importância da disponibilidade de recursos adequados para o desempenho eficaz das atividades de enfermagem.

*Se não tem material adequado para fazer um curativo, para fazer uma punção, para fazer uma sutura numa cirurgia, a assistência é prejudicada* [...]. (E04)

*Às vezes, você acaba perdendo sua punção por conta de um polifix que não encaixa com um equipo, uma seringa que quebra, então a qualidade do material é essencial para um bom trabalho.* (TE05)

*Materiais de qualidade ruim, luvas às vezes, que não tem o seu tamanho. Você usa luva tamanho p e só tem luva tamanho g. Você tem um risco ali, a questão da biossegurança também está envolvida.* (E08)

Além disso, observou-se que, frequentemente, há a ausência completa de determinados insumos, o que obriga os profissionais a improvisarem soluções para realizar o cuidado. Esse cenário gera riscos tanto para o profissional quanto para o paciente, além de resultar em maior desgaste físico e emocional, intensificando o estresse no ambiente de trabalho.

*Falta muita coisa, medicação às vezes tem, às vezes não tem. A gente não pode contar com a medicação porque tem dia que tem na instituição e tem dia que não tem.* (TE18)

[...] *então, você acaba prestando um serviço de má qualidade porque você não tem o material de acordo com a sua necessidade de trabalho. Outra coisa também é a falta de insumos, principalmente na hora do controle de infecção.* (E05)

*É a falta de material* [...] *algumas coisas básicas que ajudariam muito a gente prestar uma assistência melhor e não é visto como prioridade.* (TE03)

### Classe 2: Dimensão processual - As condições do trabalho da enfermagem como desafios à qualidade da assistência de enfermagem

Na classe 2, as falas dos profissionais de enfermagem revelam um quadro preocupante de condições de trabalho precárias que intervêm tanto na saúde desses trabalhadores quanto na qualidade da assistência prestada aos pacientes. Aspectos como ausência de apoio organizacional, excesso de carga horária e fragilidades estruturais do sistema de saúde foram pontos recorrentes que geraram insatisfação e repercussões negativas.

*Eles queriam me afastar, me recusei e continuei trabalhando assim, e essa escala acabou com a minha saúde. Nesse período engordei, fiquei obesa, desenvolvi um monte de doença, não vi minha família e quase sofri acidente várias vezes na estrada.* (TE18)

*Com certeza, as pessoas à minha volta na verdade já falam isso. Recentemente, eu tive burnout, e a melhor coisa que aconteceu para mim foi mudar de setor, porque, para mim, estava muito puxado, e aí eu não consegui. Eu mudei totalmente*. (TE03)

*Não tem como você ver um quarto sujo que não tiraram um lixo e não falar. Se você está ali, naquele meio, então você acaba sendo responsável por resolver situações que não são da sua competência.* (E08)

As falas dos profissionais destacaram a falta de suporte e apoio organizacional como um dos principais desafios enfrentados no dia a dia. Além disso, o excesso de carga horária foi identificado como um fator constante nas falas, sendo apontado como uma interferência significativa na qualidade do trabalho e na saúde dos profissionais.

*A carga horária poderia ser menor* [...] *é um pouco complicado porque a saúde hoje em dia está precária para todo o Estado, e em especial para o município.* (TE02)

[...] *a pessoa se direciona à sua chefia, e a sua chefia também, às vezes, não tem autonomia para falar para você ou para ajudar a solucionar o problema.* (TE19)

Outro ponto destacado pelos participantes da amostra é o remanejamento de profissionais entre os setores da instituição. Para os profissionais, essa dinâmica demonstra a falta de reconhecimento de suas habilidades específicas e do impacto que essas mudanças têm em sua saúde mental e no desempenho de suas funções.

*Você vem para trabalhar* [...] *eu sou do berçário, e eu venho para trabalhar no berçário. Você está no mesmo plantão, e do nada você é posto em outro setor que não tem nada a ver com o seu.* (TE02)

*A gente já vem com aquela ansiedade, e quando chega, é realmente aquilo que vai acontecer* [...] *é um estresse e ainda cobram demais da gente*. (TE09)

*Eu nem estou dizendo de falta por férias, mas sim de completar a escala. É uma coisa que a gestão atualmente não tem como resolver e a questão de infraestrutura também. A gente está falando da gestão de enfermagem.* (E10)

A desvalorização da enfermagem foi percebida pelos profissionais de enfermagem, ressaltando a falta de reconhecimento e a baixa remuneração que recebem como um fator interveniente na qualidade da assistência prestada.

*Aqui não é ruim de trabalhar. Você precisa das coisas em todas as unidades de saúde, mas o déficit de funcionários, essa carga horária de ter que ficar trabalhando em outros lugares, porque nem o piso salarial eles conseguem pagar.* (E06)

*A valorização do profissional de enfermagem é muito ruim. Muitas vezes, você está sem paciência com aquele paciente, porque tem pessoas que misturam os problemas.* (TE19)

[...] *a carga de trabalho na enfermagem, A gente precisa trabalhar em vários lugares. O problema não é só isso, é o acúmulo de trabalho devido ao salário que é baixo.* (TE12)

### Classe 3: Dimensão resultado - A sobrecarga e precarização do cuidado como fatores intervenientes na qualidade da assistência de enfermagem

Na classe 3, os relatos dos profissionais de enfermagem destacam o subdimensionamento de pessoal como um fator interveniente crucial na qualidade da assistência prestada. A insuficiência de trabalhadores, apontada pelos participantes, sobrecarrega a equipe, aumentando a carga de trabalho e comprometendo a capacidade de fornecer cuidados humanizados e seguros. Além disso, o remanejamento de profissionais entre os setores é visto como um fator crítico que desestabiliza as equipes, prejudica a continuidade do cuidado e interfere diretamente na organização do trabalho.

*Então, às vezes, tira a técnica daqui para suprir um lugar por conta do horário de descanso do outro setor, para poder fazer o descanso. A gente fica com pacientes psiquiátricos e sem quantitativo de profissionais, e isso atrapalha a assistência.* (TE08)

*A quantidade de técnicos atuantes, eu acho que é inferior à quantidade de pacientes que a gente recebe no nosso setor. Por exemplo, aqui é a emergência* [...]. (E09)

*Trabalhar um enfermeiro e um técnico para uma demanda alta de pacientes. Acho que o número de pessoal interfere diretamente na qualidade da assistência, porque você não tem como estar em todos os lugares ao mesmo tempo.* (TE13)

*O número de funcionários é muito reduzido, e isso acaba gerando uma sobrecarga na função deles e acaba prejudicando a assistência. A gente acaba não dando uma assistência de qualidade. Se ele está sobrecarregado, vai pesar na qualidade da assistência e no prognóstico do paciente. Isso tudo vai interferir na assistência de qualidade que a gente presta.* (E04)

A falta de horários adequados para o descanso emerge como um problema recorrente, conforme relatado pelos profissionais de enfermagem, que frequentemente destacam a dificuldade de realizar pausas regulares durante os turnos de trabalho. Nas falas, ficou evidente que a ausência de descanso impacta negativamente a capacidade de concentração e a tomada de decisão dos profissionais, resultando em um cuidado mais vulnerável e potencialmente inseguro para os pacientes.

*Mas para você tirar sua hora de descanso, você tem que ter um espaço legal para tomar um banho e não tem. Nem sempre você vai tirar um tempo para dormir.* (TE16)

*No horário da medicação da meia-noite, eu já não tenho a capacidade de saber nem o que eu estou fazendo mais, porque foi desde oito horas da manhã até meia-noite sem parar o plantão, sem fazer hora de almoço e às vezes sem descanso. Só com dois técnicos no setor.* (TE13)

*Porque a gente estava com apenas um técnico no setor, uma tenda com 12 pacientes nos leitos. Paciente acamados com sonda enteral ou sonda vesical* […] *aí a gente teve que priorizar a medicação e sinais vitais.* (E07)

[...] *para esse horário de descanso, acho que isso seria fundamental, tanto para o nosso psicológico quanto para a gente poder descansar e a gente não ter um horário para descansar. A gente fica cansado, e esse cansaço às vezes atrapalha.* (TE08)

## DISCUSSÃO

Os achados deste estudo revelaram distintos fatores intervenientes relacionados às condições de trabalho na qualidade da assistência de enfermagem, entre os quais destacaram-se insuficiência de recursos materiais, materiais de baixa qualidade, subdimensionamento de pessoal, falta de tempo para descanso, remanejamento de pessoal, sobrecarga de trabalho e falta de apoio organizacional. De modo direto, a ausência de insumos básicos e materiais de baixa qualidade foi percebida como fator interveniente na qualidade da assistência de enfermagem, uma vez que os profissionais participantes recorrem à adoção de soluções improvisadas que podem resultar em erros na prática profissional e comprometer a segurança da assistência, de acordo com as falas.

Estudo realizado em dois hospitais gerais do Irã, em Teerã, que envolveu 211 enfermeiros com pelo menos dois anos de experiência clínica, apontou a inadequação de recursos como um fator preditivo significativo para infecções associadas ao cuidado de saúde e que eleva os níveis de estresse dos profissionais, aumentando a sensação de impotência, dados que corroboram os achados atuais^(20)^. Outros estudos também estão em consonância com os achados deste estudo, evidenciando a relação direta entre a falta de materiais, a elevada carga de trabalho e as condições precárias enfrentadas por profissionais de enfermagem^(20,21)^.

Além da insuficiência de insumos, a carga horária excessiva, a sobrecarga de trabalho e o subdimensionamento de pessoal de enfermagem se apresentaram como fatores intervenientes à qualidade da assistência de forma indireta, uma vez que os profissionais cansados, sobrecarregados e com déficit de pessoal na equipe não conseguem atender às demandas de trabalho, prejudicando sua relação profissional-paciente. Estudo em seis Unidades de Cuidados Intensivos com 105 enfermeiros mostrou que longas jornadas, acúmulo de funções e sobrecarga diminuem o tempo para a prestação de cuidados, elevando o risco de erros, como administração incorreta de medicamentos, quedas de pacientes e infecções hospitalares^(22)^, assim como foi mencionado pelos participantes desta pesquisa.

Da mesma maneira, pesquisa realizada entre 810 enfermeiras hospitalares nos Estados Unidos da América, com jornada de pelo menos 36 horas semanais, revelou que a sobrecarga de trabalho compromete a saúde dos profissionais e a segurança do paciente, destacando a necessidade de intervenções organizacionais para melhorar a alocação de pessoal e distribuição de funções^(23)^. O subdimensionamento de pessoal de enfermagem compromete a eficiência técnica, as responsabilidades éticas dos profissionais e a qualidade da assistência. Estudo nas Filipinas apontou que a sobrecarga de trabalho e a falta de recursos agravam o problema, exigindo reformas estruturais no sistema de saúde. A alta proporção de pacientes por enfermeiro intensifica a sobrecarga, contribuindo para insatisfação, esgotamento e eventos adversos, além de reduzir a satisfação dos profissionais^(21)^. Tal cenário é similar ao percebido pelos participantes desta pesquisa.

A insuficiência de profissionais resulta em uma sobrecarga de trabalho que frequentemente leva à omissão de cuidados essenciais. De acordo com a Resolução COFEN nº 543/2017, o dimensionamento adequado é indispensável para garantir uma assistência segura e de qualidade, sendo uma atribuição exclusiva do enfermeiro^(24)^. Os resultados de investigação realizada em três Unidades de Terapia Intensiva de hospitais no Brasil com 29 profissionais de enfermagem vão ao encontro dos achados do presente estudo, apontando que o subdimensionamento gera diversas repercussões, inclusive impactos negativos na qualidade do cuidado prestado e na satisfação dos trabalhadores^(25)^. Diante disso, dimensionar adequadamente a equipe de enfermagem é fundamental para equilibrar a carga de trabalho e melhorar as condições laborais.

Os achados desta pesquisa destacam que a falta de descanso, somada à carga horária extensa, sobrecarga de trabalho e subdimensionamento de pessoal, intervém na qualidade da assistência. O descanso dos profissionais é um direito constitucional, garantindo ao menos uma hora para jornadas acima de seis horas, duas horas, para jornadas de 12 horas e quatro horas, para jornadas de 24 horas, com possibilidade de fracionamento, conforme acordo coletivo^(26)^. É essencial, portanto, que as condições de descanso previstas em lei sejam cumpridas, com locais adequados, climatizados, isolados acusticamente e proporcionais ao número de profissionais, tanto em instituições públicas quanto em privadas, garantindo conforto, dignidade e reduzindo os impactos de jornadas prolongadas^(27)^.

A falta de descanso entre profissionais de enfermagem contribui para eventos adversos na saúde. Estudo com 588 enfermeiras na China mostrou que a má qualidade do sono e a falta de descanso adequado comprometem o julgamento, memória e atenção, aumentando o risco de erros em ambientes de alta concentração e decisões rápidas, especialmente em ambientes que exigem alta concentração e decisões rápidas, como nos serviços de saúde^(28)^.

Além do descanso, outro fator mencionado com frequência foi o remanejamento de pessoal de enfermagem, que se faz recorrente na instituição, diante do subdimensionamento. Os profissionais de enfermagem se sentem desvalorizados, o que gera insatisfação e afeta a qualidade da assistência. Problemas estruturais, baixos salários e falta de reconhecimento contribuem para a alta rotatividade e o enfraquecimento da resiliência. Assim, a percepção de desvalorização, ligada à falta de autonomia, reforça a necessidade de maior suporte da gestão para fortalecer a equipe^(29)^.

A falta de apoio organizacional e a ausência de diálogo com lideranças foram aspectos amplamente citados como agravantes das condições de trabalho. Tal fato, assim como ocorre na presente pesquisa, também foi relatado em estudo realizado em hospitais gerais do Irã, evidenciando que a insuficiência de recursos, combinada com uma liderança deficiente e a exclusão dos enfermeiros das decisões organizacionais, compromete tanto a satisfação dos profissionais quanto a qualidade do cuidado, tornando o ambiente desgastante^(20)^.

Nesse sentido, destaca-se que o apoio organizacional é crucial para lidar com a escassez de recursos humanos, materiais e insumos, além de melhorar os fluxos de trabalho e as relações interpessoais^(29)^. A ausência desse apoio se reflete em insatisfação, sobrecarga de trabalho e aumento de absenteísmo, prejudicando a assistência prestada e a motivação das equipes. O apoio organizacional é mencionado como um aspecto central para a motivação, desempenho e qualidade dos serviços prestados, além de impactar diretamente a satisfação dos trabalhadores.

### Limitações do estudo

As limitações estão associadas à natureza sensível do tema, que gerou diversas recusas à participação dos profissionais de enfermagem. Essa resistência pode ter sido influenciada pela preocupação com o sigilo e pela percepção de possíveis repercussões nas condições de trabalho e nas relações com a gestão. Apesar disso, foi possível alcançar a saturação teórica dos dados, com uma amostra representativa dos profissionais de enfermagem que atuam no cenário investigado. Ademais, a coleta de dados se restringiu ao ambiente de um único hospital público, o que pode restringir a generalização dos achados para outros contextos e instituições de saúde.

### Contribuições para as áreas da enfermagem, saúde ou políticas públicas

No ambiente hospitalar, o apoio institucional e das lideranças de enfermagem é crucial para enfrentar a escassez de recursos, melhorar relações interpessoais e otimizar fluxos de trabalho. Este estudo destaca a urgência de mudanças estruturais no sistema de saúde para proteger a saúde de profissionais e pacientes. Medidas como fiscalização do conselho regulador, pagamento do piso salarial, dimensionamento adequado de pessoal, garantia de descanso e fortalecimento das lideranças são essenciais para reverter esse cenário. Entende-se, portanto, que o bem-estar dos profissionais de enfermagem é indispensável para garantir não apenas a segurança e a qualidade da assistência, mas também a sustentabilidade do sistema de saúde. Esses esforços, quando incorporados de forma efetiva, podem transformar significativamente a qualidade do cuidado, os desfechos clínicos e a experiência dos profissionais no ambiente hospitalar.

## CONSIDERAÇÕES FINAIS

As condições de trabalho, como ausência de períodos e locais adequados para descanso, o remanejamento frequente para outros setores, os baixos salários, a desvalorização profissional e a falta de apoio institucional interferem diretamente na qualidade da assistência de enfermagem. Além disso, os profissionais de enfermagem percebem como fatores intervenientes a escassez de materiais e insumos, a sobrecarga de trabalho, e o subdimensionamento da equipe.

O estudo apresenta importantes contribuições para a prática de enfermagem ao gerar reflexões críticas entre profissionais líderes e gestores, no sentido de subsidiar estratégias que favoreçam o aprimoramento das condições laborais e, consequentemente, da qualidade da assistência prestada. Portanto, os resultados apontam para a necessidade de implementação de mudanças organizacionais que promovam a valorização dos profissionais, garantam o bem-estar da equipe e assegurem condições adequadas de trabalho. Nesse sentido, estratégias viáveis para fortalecer o apoio organizacional, aprimorar a comunicação interna e fomentar um ambiente colaborativo incluem a realização de reuniões periódicas, rodas de escuta e momentos de interlocução coletiva. Essas ações podem funcionar como ferramentas para o mapeamento contínuo do cenário institucional, fornecendo subsídios concretos para a formulação e implementação de mudanças coerentes com a realidade dos serviços.

Além disso, o estudo ressalta a responsabilidade do Estado como empregador, gestor e regulador dos serviços públicos de saúde, o qual deve assegurar condições adequadas de trabalho às equipes. A omissão nesse papel contribui para a perpetuação de problemas estruturais e humanos que comprometem não apenas a qualidade da assistência, mas também a saúde física e mental dos trabalhadores de enfermagem, repercutindo diretamente na segurança do paciente. Assim, os achados do estudo fornecem evidências que podem subsidiar políticas públicas voltadas à reestruturação do trabalho em enfermagem, à valorização profissional e ao fortalecimento da assistência, a fim de promover transformações sustentáveis que assegurem a qualidade do serviço ofertado e a saúde dos trabalhadores.

Por fim, sugere-se ainda que futuras pesquisas de implementação sejam realizadas, especialmente na área da gestão de enfermagem, com foco na avaliação de indicadores relacionados às condições de trabalho antes e após a adoção de intervenções institucionais prioritárias. Estudos que considerem a participação ativa dos trabalhadores e incluam a análise do impacto das mudanças adotadas poderão fortalecer a aplicabilidade dos achados e contribuir para a melhoria contínua da qualidade da assistência de enfermagem e da organização do trabalho nos serviços de saúde.

## Data Availability

Os dados de pesquisa só estão disponíveis mediante solicitação.
